# Endoglin and Activin Receptor-like Kinase 1 (Alk1) Modify Adrenomedullin Expression in an Organ-Specific Manner in Mice

**DOI:** 10.3390/biology11030358

**Published:** 2022-02-24

**Authors:** Josune García-Sanmartín, Judit Narro-Íñiguez, Alicia Rodríguez-Barbero, Alfredo Martínez

**Affiliations:** 1 Angiogenesis Unit, Oncology Area, Center for Biomedical Research of La Rioja (CIBIR), 26006 Logrono, Spain; jgarcias@riojasalud.es (J.G.-S.); jnarro@riojasalud.es (J.N.-Í.); 2 Vascular Endothelium Pathophysiology (ENDOVAS) Unit, Department of Physiology and Pharmacology, University of Salamanca, 37007 Salamanca, Spain; barberoa@usal.es; 3 Biomedical Research Institute of Salamanca (IBSAL), 37007 Salamanca, Spain

**Keywords:** adrenomedullin, hereditary hemorrhagic telangiectasia (HHT), endoglin, ALK1, BMP-9

## Abstract

**Simple Summary:**

Hereditary hemorrhagic telangiectasia (HHT) is called a rare disease because it affects relatively few people. It is characterized by malformations in some blood vessels and usually results in profuse nose bleedings. In a recent article, we found that these patients have higher levels of adrenomedullin (AM), a molecule with cardiovascular activities, than healthy people. Thus we wanted to know whether the mutations that cause the HHT disease are directly responsible for these higher levels of AM. To investigate this issue, we used mutant mice, which express lower levels of the genes involved in the disease (called *Eng* and *Acvrl1*), and measured how much AM was found in different tissues. Although we expected a higher amount of AM in all organs, that was not the case. Some organs showed no variation, some had lower levels of AM than normal mice (fat, skin, and adrenals), and others had a higher expression (cerebellum and colon). Interestingly, our results suggest that these genes and the related molecule BMP-9 may have novel functions, which have not been yet investigated, which may shed more light on the physiopathology of HHT.

**Abstract:**

Hereditary hemorrhagic telangiectasia (HHT) is a rare disease characterized by vascular malformations and profuse bleeding. The disease is caused by mutations in the components of the BMP-9 receptor: endoglin (*ENG*) and activin receptor-like kinase 1 (*ACVRL1*) genes. Recently, we reported that HHT patients expressed higher serum levels of adrenomedullin (AM) than healthy volunteers; thus, we studied the expression of AM (by enzyme immunoassay, qRT-PCR, immunohistochemistry, and Western blotting) in mice deficient in either one of the receptor components to investigate whether these defects may be the cause of that elevated AM in patients. We found that AM expression is not affected by these mutations in a consistent pattern. On the contrary, in some organs (blood, lungs, stomach, pancreas, heart, kidneys, ovaries, brain cortex, hippocampus, foot skin, and microvessels), there were no significant changes, whereas in others we found either a reduced expression (fat, skin, and adrenals) or an enhanced production of AM (cerebellum and colon). These results contradict our initial hypothesis that the increased AM expression found in HHT patients may be due directly to the mutations, but open intriguing questions about the potential phenotypic manifestations of *Eng* and *Acvrl1* mutants that have not yet been studied and that may offer, in the future, a new focus for research on HHT.

## 1. Introduction

Hereditary hemorrhagic telangiectasia (HHT) is a rare disease characterized by telangiectases and larger vascular malformations of the pulmonary, cerebral, or hepatic vasculature [1]. Telangiectasis is an abnormal communication between an arteriole and a dilated and tortuous venule in the capillary bed. HHT can be diagnosed either through molecular genetic testing or using the Curaçao clinical criteria (recurrent epistaxis, cutaneous/mucosal telangiectasia, visceral vascular malformations, and a first-degree family member with HHT) [2]. Mutations in the endoglin (*ENG*) and activin A receptor type II-like 1 (*ACVRL1*) genes are detected in approximately 90% of cases and cause HHT1 and HHT2, respectively [3].

Activin receptor-like kinase 1 (ALK1; encoded by *ACVRL1*) is a type I receptor for the transforming growth factor β (TGFβ)/bone morphogenetic protein 9 (BMP-9) signaling pathway. Endoglin (CD105; encoded by *ENG*) is a co-receptor, which, together with ALK1, constitutes the functional receptor for BMP-9. Both receptor components are located at the endothelial cell membrane. The activation of the BMP-9 receptor induces Smad signaling, which promotes endothelial cell proliferation, migration, and survival during angiogenesis. Loss of either endoglin or ALK1 function provokes anomalous vascular overgrowth due to the overactivation of phosphatidylinositol 3-kinase (PI3K) signaling [4]. Although endoglin and ALK1 are components of the same BMP-9 receptor complex, the pathogenic variants in their genes are related to different clinical phenotypes [5].

Animal models where the genes for either endoglin [6] or ALK1 [7] were knocked out have been generated to try to better understand the human disease. Homozygous mutants for either gene die in utero due to gross vascular malformations, but the heterozygotes lead a rather healthy life, despite some vascular dysfunctions [8,9]. Surprisingly, no telangiectases are ever found in haploinsufficient mice, indicating that this HHT hallmark requires further genetic triggers or is just a specifically human characteristic.

Recently, we reported that HHT patients present higher serum levels of the cardiovascular regulatory peptide adrenomedullin (AM) than healthy volunteers, and that immunoreactivity for this peptide accumulates in the abnormal blood vessels of the telangiectases [10]. AM is a 52 amino acid peptide hormone that belongs to the amylin/calcitonin gene-related peptide family [11]. AM is synthesized as part of a larger precursor molecule, named pre-pro-AM, and is secreted by many cells/tissues, including vascular endothelial and smooth muscle cells [12]. This hormone has multiple actions that are exerted through combinations of the calcitonin receptor-like receptor (CLR), and either receptor activity-modifying protein 2 (RAMP2) or RAMP3, also known as AM1 and AM2 receptors, respectively [13]. AM plays critical roles in blood vessels, having vasodilatory properties and helping to regulate vascular stability and permeability by modulating the endothelial barrier [14,15]. Moreover, it participates in regulating circulatory homeostasis and in the pathogenesis of certain cardiovascular and cerebrovascular diseases [16,17], and has an ample distribution in all tissues and organs [16].

Our goal for this study was to investigate whether the elevated levels of AM found in HHT patients are a consequence of the mutations on the BMP-9 receptor. To achieve this, we used molecular and immunohistological techniques to measure AM expression in mice with reduced expression of either receptor component.

## 2. Materials and Methods

### 2.1. Animals and Tissue Collection

All animal protocols were performed with the approval of the committee on bioethics of the University of Salamanca (protocol 305) and complied with the current guides of the European Union and the US Department of Health and Human Services for the Care and Use of Laboratory Animals. The generation of *Eng^+/−^* and *Alk1^+/−^* mice has been described previously [6,18]. The animals were a generous gift from Michelle Letarte (Hospital for Sick Children, Toronto, Canada) and Peter ten Dijke (Leiden University, Netherlands). The animals were housed under specific pathogen-free conditions at the University of Salamanca’s facilities (ES-119-002001 SEARMG) in a temperature-controlled room with a 12 h light/dark cycle and reared on standard chow and water provided *ad libitum*. Routine genotyping of DNA isolated from mouse tail biopsies was performed by PCR using the primers previously reported [18,19]. *WT* littermates for each model were used as controls. Four *Eng^+/−^*, four *Alk1^+/−^*, and eight *WT* animals were sacrificed by an anesthetic overdose (pentobarbital sodium, Dolethal, Lure, France), blood was collected through a cardiac puncture, and different organs were immediately dissected out and divided into small fragments that were either frozen in liquid nitrogen and stored at −80 °C for RNA extraction, or fixed in 10% buffered formalin and paraffin embedded for immunohistochemical studies.

### 2.2. Chemiluminescent Enzyme Immunoassay (EIA)

The blood serum concentrations of AM were determined in triplicate using a commercially available EIA kit (CEK-010-08, Phoenix Pharmaceuticals, Karlsruhe, Germany). Samples (0.5 mL) were initially diluted in an equal volume of 0.1% alkali-treated casein (ATC) in phosphate-buffered saline (PBS), pH 7.4, and applied to pre-washed reverse-phase Sep-Pak C18 cartridges (Waters Corp., Milford, MA, USA). The peptide fraction was eluted from the C18 matrix with 3 mL 80% isopropanol containing 0.125N HCl and freeze–dried overnight, as previously described [20]. The AM levels in lyophilized extracts were then determined by EIA following the manufacturer’s instructions. Chemiluminescence was measured in a plate reader (POLARstar Omega, BMG Labtech, Ortenberg, Germany). This procedure has an intra-assay variation lower than 10% and an inter-assay variation lower than 15%.

### 2.3. RNA Extraction, Reverse Transcription, and Real Time Polymerase Chain Reaction

Total RNA was isolated from mouse tissues and purified as described [21] using Trizol reagent (Invitrogen, Waltham, MA, USA) with a DNAse digestion step performed (Qiagen, Germantown, MD, USA) according to the manufacturer’s instructions. The resulting RNA (5 µg) was reverse-transcribed using Superscript III First-Strand Synthesis System for RT-PCR (Invitrogen), and the synthesized cDNA was amplified using SYBR Green PCR Master Mix (Applied Biosystems, Bedford, MA, USA). The transcripts were amplified by real-time PCR (QuantStudio 5, Applied Biosystems). A specific calibration curve of cDNA was included to analyze the expression of the mouse adrenomedullin (*Adm*) gene and of *Gapdh* as a housekeeping gene (Table 1).

### 2.4. Immunohistochemistry

Tissue sections (3 µm-thick) were dewaxed in xylene and endogenous peroxidase was blocked with 3% H_2_O_2_ in methanol for 15 min. The samples were rehydrated and subjected to antigen retrieval (10 mM Sodium Citrate, 0.5% Tween 20, pH 6.0, 20 min at 95 °C). Non-specific binding was blocked by exposure to Protein block buffer (Novocastra Leica Biosystems, Newcastle, UK) for 30 min. Then, the tissue sections were incubated with rabbit polyclonal antibody against human AM (ab69117, Abcam, Cambridge, UK), at 1:3000 dilution and 4 °C overnight. The following day, the sections were incubated with Novolink Polymer (Novocastra Leica Biosystems, Wetzlar, Germany), followed by exposure to 3,3′-diaminobenzidine (Dako, Carpinteria, CA, USA). The slides were lightly counterstained with hematoxylin and analyzed with an Eclipse 50i microscope (Nikon, Tokyo, Japan) equipped with a DXM 1200c digital camera (Nikon). The substitution of the primary antibody by PBS and liquid- or solid-phase preabsorption of the antibody [22,23] in serial sections were used as negative controls. Briefly, liquid- and solid-phase absorption controls were performed with synthetic AM (Phoenix Pharmaceuticals). For liquid-phase absorption, 20 nmol AM was added per mL of optimally diluted antibody and incubated overnight at 4 °C and tissue sections were then incubated in the resulting mix. For solid-phase absorption, polystyrene tubes (VWR International, Radnor, PA, USA) were coated with 20 nmol/mL AM for 2 h rotating at room temperature, blocked with 1% BSA in PBS for 2 h at room temperature, and then incubated with the optimally diluted antibody overnight at 4 °C. Then, the pre-absorbed antibody was added to the sections. In parallel, antibodies pre-absorbed in BSA-coated tubes were used as a positive control.

### 2.5. Quantification of Immunohistochemical Signals

At least three stained sections of each experimental group and organ were analyzed. Care was taken in selecting anatomically matched areas among animals before the assays. Immunoreactivity was evaluated using the ImageJ free software (NIH, Bethesda, MD, USA), following the published guidelines [24]. The procedure included the selection of the region of interest, color deconvolution, threshold setting, and the measurement of the fraction area (percentage of pixels highlighted in red from the selected area).

### 2.6. Western Blotting

Tissues were lysed in Pierce RIPA Buffer (Invitrogen) supplemented with Complete Protease Inhibitor Cocktail (Roche Diagnostics, Basel, Switzerland). The protein content of the lysates was quantified using the Bradford Protein Assay (BioRad, Hercules, CA, USA). Nupage sample reducing buffer (Invitrogen) was added to the lysates (50 µg), heated at 70 °C for 10 min, and separated by SDS polyacrylamide gel electrophoresis in 4–12% Bis-Tris gels (Invitrogen). Electrophoresed proteins were transferred to PVDF membranes using the iBlot 2 Transfer Device (Invitrogen). Once transferred, the membranes were blocked using 0.5% (*w*/*v*) ATC in Tris-buffered saline–Tween-20 (TBST; 25 mM Tris, pH 7.5, 150 mM NaCl, 0.1% (*v*/*v*) Tween-20) and then cut into two halves through the 30 kDa weight standard. The half of the membrane containing low-molecular-weight proteins was exposed overnight to the rabbit anti-AM antibody at a 1:2000 dilution, and the high-molecular-weight half to mouse monoclonal anti-GAPDH antibody (ab8245, Abcam) at a 1:10,000 dilution. On the next day, horseradish peroxidase (HRP)-linked secondary antibodies to rabbit (7074, Cell Signaling, Danvers, MA, USA) or mouse IgGs (715-035-151, Jackson Immunoresearch, West Grove, PA, USA) were used at a 1:12,000 and 1:50,000 dilutions, respectively. The peroxidase activity was detected with ECL Prime Western Blotting Detection Reagent (Cytiva, Marlborough, MA, USA) and captured in a ChemiDoc MP Imaging System (BioRad). The quantification of the immunoreactive bands was accomplished with the ImageLab software (BioRad).

### 2.7. Statistical Analysis

The normality of the dataset distribution was assessed using the one-sample Kolmogorov–Smirnov test. Since the number of data was small and the distribution was not normal, all datasets were compared with the Kruskal–Wallis one-way ANOVA test. A two-sided *p*-value lower than 0.05 was considered statistically significant. Analyses were performed using Prism, version 8.3.0 (GraphPad software, San Diego, CA, USA).

## 3. Results

Four endoglin heterozygous mice (*Eng^+/−^*), four Alk1 heterozygous mice (*Alk1^+/−^*), and eight *WT* controls were sacrificed, their blood was taken, and their tissues were either fixed in formalin and paraffin-embedded for immunohistochemistry or frozen in liquid N_2_ for molecular analysis.

### 3.1. AM Blood Levels

**Figure 1:**
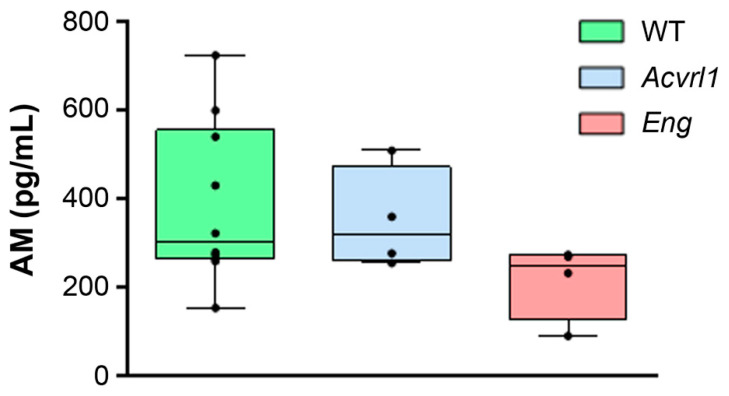
Circulating levels of AM in the serum of mice belonging to the three genotypes measured by EIA. Box plots represent the interquartile range with the median as the horizontal line. Whiskers encompass the maximum and minimum values of the population. Dots represent individual animals.

The serum AM levels were measured by EIA in samples from the three genotypes. The median (Q1–Q3) serum AM levels were 300.3 (262.0–554.6) pg/mL for WT animals, 317.8 (260.0–470.8) for *Alk1^+/−^*, and 250.0 (125.0–271.8) for *Eng^+/−^* mice. No significant differences were found when comparing the groups (Figure 1).

### 3.2. Immunohistochemical Controls

**Figure 2:**
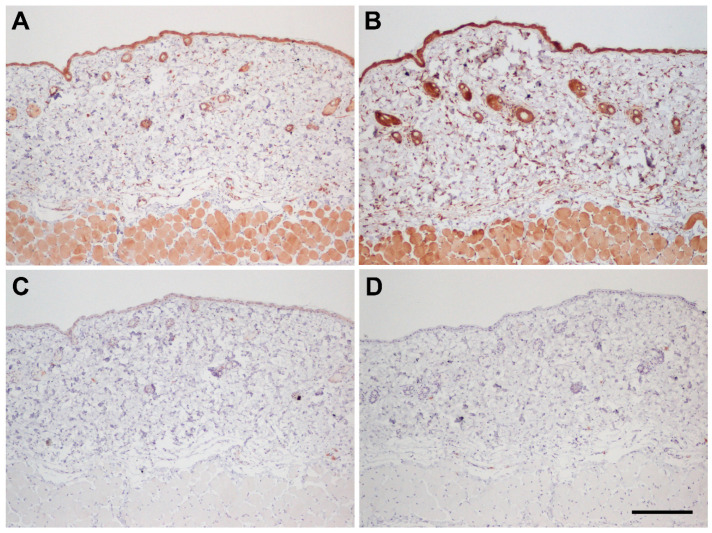
Serial sections of the dorsal skin of a *WT* mouse showing specificity controls for the AM antibody. The regular staining protocol shows the AM immunoreactivity in the epithelium and associated glands, hair follicles, blood vessels, as well as in the striated muscle of the dermis (**A**). Preabsorption with synthetic peptide in the liquid phase resulted in a paradoxical staining increase (**B**). Solid phase preabsorption with synthetic peptide completely prevented staining (**C**). Suppression of the primary antibody also prevented labeling (**D**). Scale bar for (**A**–**D**) = 200 µm.

The antibody used for immunohistochemistry has been employed before in human tissues [10], but has not been tested in mice before. Therefore, before starting the immunohistochemical study, we characterized the specificity of this antibody in mouse tissues. The first test was the application of the primary antibody pre-absorbed with synthetic peptide in the liquid phase. Interestingly, this procedure resulted in a paradoxical staining increase (Figure 2B) compared to the BSA-preabsorbed antibody (Figure 2A). This has been previously reported for other antibodies and usually indicates a specific antigen–antibody binding [22,23]. Then, we tested the solid-phase absorption of the antibody, and this produced the desired quenching of the staining (Figure 2C), demonstrating antibody specificity. In addition, we tested primary antibody omission. This maneuver resulted in a lack of labeling (Figure 2D), thus showing that all other layers of the immunohistochemical procedure (polymer, peroxidase, etc.) did not contribute to the staining.

### 3.3. Expression of AM in Organs of Endodermic Origin

**Figure 3:**
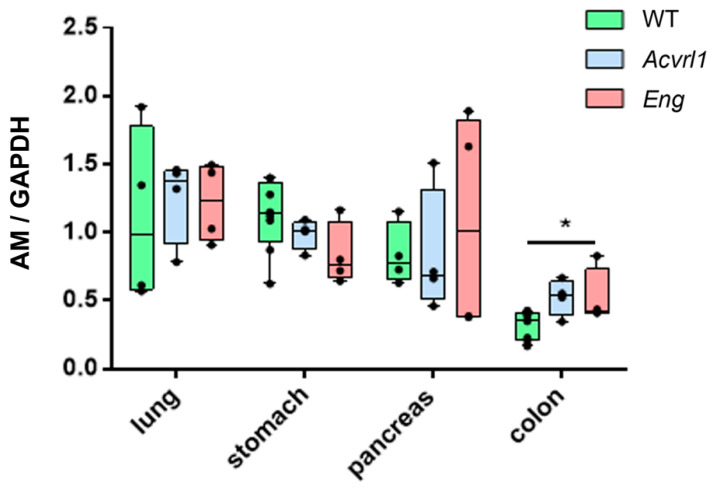
AM expression in endoderm-derived organs as measured by qRT-PCR. No differences were found among genotypes in the lungs, stomach, or pancreas. Box plots represent the interquartile range with the median as a horizontal line. Whiskers encompass the maximum and minimum values of the population. Dots represent individual animals. *: *p* < 0.05.

**Figure 4:**
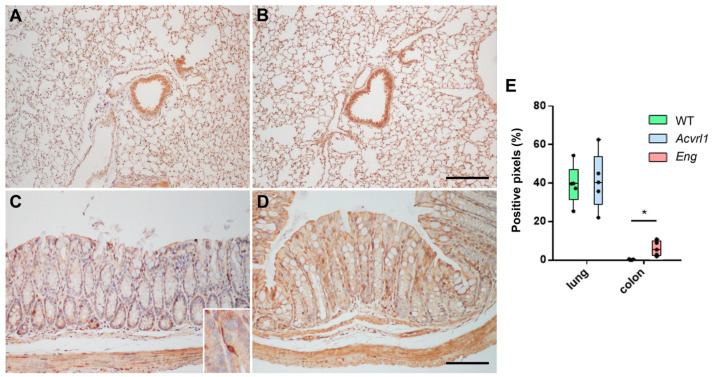
Representative photographs of the lung (**A**,**B**) and colon (**C**,**D**) of *WT* animals (**A**,**C**), and mutants for *Acvrl1* (**B**) and *Eng* (**D**), stained with the anti-AM antibody. The inset in C shows a higher magnification of a cell from the colonic endocrine diffuse system. (**E**) Quantification of the immunohistochemical signal. Box plots represent the interquartile range with the median as a horizontal line. Whiskers encompass the maximum and minimum values of the population. Dots represent individual animals. *: *p* < 0.05. Scale bar for (**A**,**B**) = 200 µm and (**C**,**D**) = 100 µm.

AM expression did not change in the lungs of mutant mice. This was confirmed at the molecular (Figure 3) and immunohistochemical levels (Figure 4A,B). AM was mainly expressed in the smooth muscle cells of the bronchioles and blood vessels, and in the bronchiolar epithelium (Figure 4A,B). In the digestive system, there were no changes in the upper part, including the stomach and pancreas, but significant changes were shown in the colon, where a higher expression of AM was observed in mutant *Eng* mice (*p* = 0.028) (Figure 3). In the colon, AM was mainly found in the apical membrane of the epithelium and in cells of the diffuse endocrine system (Figure 4C,D). As in the lungs, the smooth muscle cells of the muscle layers and blood vessels were also positive for AM (Figure 4C,D). The quantification of the immunoreactivity showed a significant increase in the colon of mutant mice (*p* = 0.025) (Figure 4E).

### 3.4. Expression of AM in Organs of Mesodermic Origin

**Figure 5:**
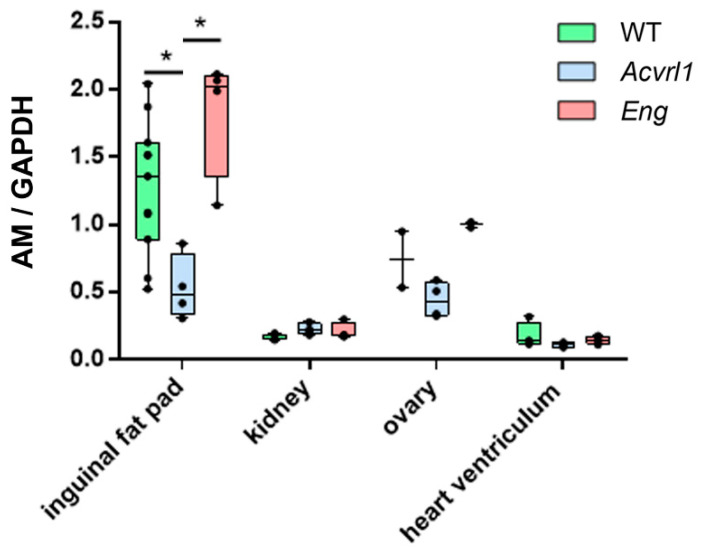
AM expression in mesoderm-derived organs as measured by qRT-PCR. Box plots represent the interquartile range with the median as a horizontal line. Whiskers encompass the maximum and minimum values of the population. Dots represent individual animals. *: *p* < 0.05.

Several organs derived from the mesodermic layer were studied, including the heart, kidneys, ovaries, and inguinal fat. No significant changes in AM expression were observed in the heart, kidneys, and ovaries (Figure 5). In contrast, mutant mice for *Acvrl1* had significantly lower levels of AM in their inguinal fat than either the *WT* or the *Eng* mutants (*p* = 0.010 and *p* = 0.028, respectively) (Figure 5).

### 3.5. Expression of AM in Organs of Ectodermic Origin

**Figure 6:**
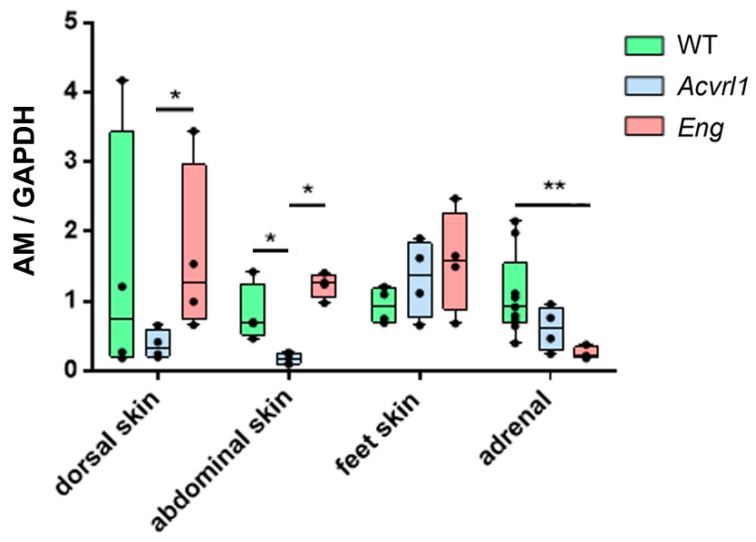
AM expression in some ectoderm-derived organs as measured by qRT-PCR. Box plots represent the interquartile range with the median as a horizontal line. Whiskers encompass the maximum and minimum values of the population. Dots represent individual animals. *: *p* < 0.05; **: *p* < 0.01.

**Figure 7:**
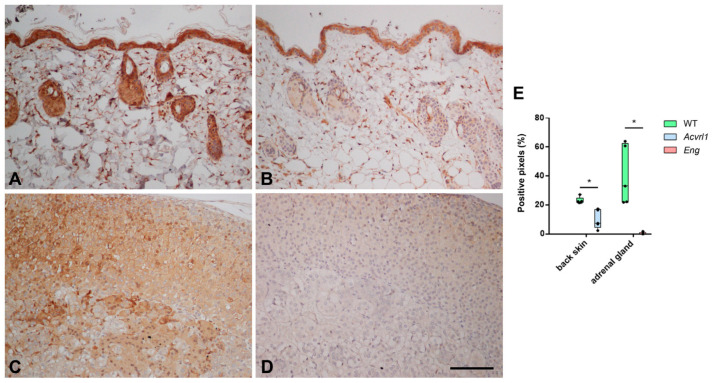
Representative photographs of the dorsal skin (**A**,**B**) and adrenal glands (**C**,**D**) of *WT* animals (**A**,**C**), and mutants for *Acvrl1* (**B**) and *Eng* (**D**) stained with the anti-AM antibody. (**E**) Quantification of the immunohistochemical signal. Box plots represent the interquartile range with the median as a horizontal line. Whiskers encompass the maximum and minimum values of the population. Dots represent individual animals. *: *p* < 0.05. Scale bar for (**A**–**D**) = 100 µm.

**Figure 8:**
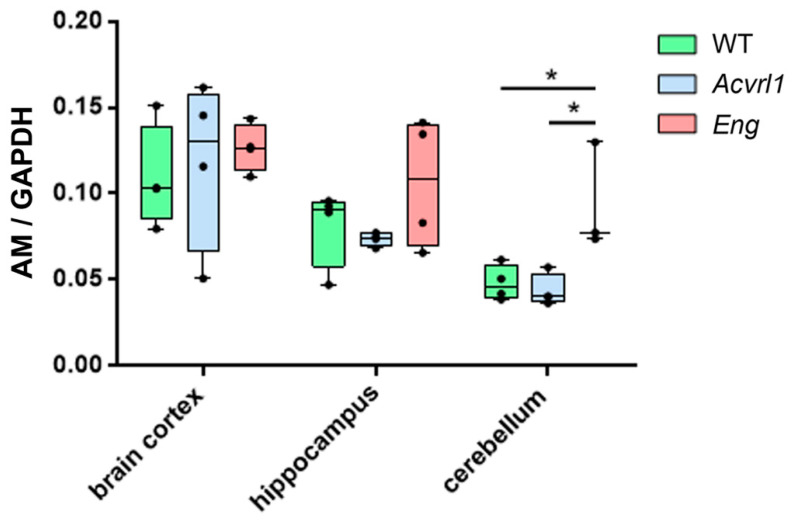
AM expression in the organs of the central nervous system as measured by qRT-PCR. Box plots represent the interquartile range with the median as a horizontal line. Whiskers encompass the maximum and minimum values of the population. Dots represent individual animals. *: *p* < 0.05.

In the skin, a regular target of HHT, there was a lower expression of AM in *Acvrl1* mice compared with either the *WT* or the *Eng* mutants when studying dorsal and abdominal skin, but there were no differences in the skin of the feet (Figure 6). In the skin, AM was mainly expressed by the epithelium, associated glands, and hair follicles (Figure 7A,B,E). In addition, we found an intense reduction in AM in the adrenals of the *Eng* mutants (*p* = 0.003) (Figure 6 and Figure 7C,D). In this gland, which provides its name to AM, AM immunoreactivity was found both in the medulla and in the cortex (Figure 7C–E). In the central nervous system, we found no changes in the cerebral cortex and the hippocampus, but there was a significant increase in AM expression in the cerebellum of *Eng* mutants over *WT* (*p* = 0.028) and *Acvrl1* mice (*p* = 0.028) (Figure 8).

### 3.6. Expression of AM in Blood Vessels

**Figure 9:**
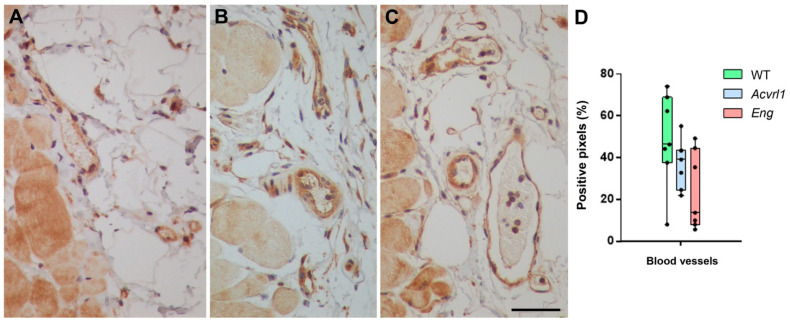
Representative photographs of small blood vessels in the dorsal skin of *WT* animals (**A**) and mutants for *Acvrl1* (**B**) and *Eng* (**C**), stained with the anti-AM antibody. The endothelium and the smooth muscle cells appear clearly stained. (**D**) Quantification of the immunohistochemical signal. Box plots represent the interquartile range with the median as a horizontal line. Whiskers encompass the maximum and minimum values of the population. Dots represent individual animals. Scale bar for (**A**–**C**) = 50 µm.

HHT is a vascular disease characterized by arterio-venous telangiectases. Therefore, we paid special attention to the AM staining of blood vessels in all organs. AM immunoreactivity was found in the endothelium and the smooth muscle cells of the blood vessels, but no evident differences were found among genotypes (Figure 9).

### 3.7. Western Blots

**Figure 10:**
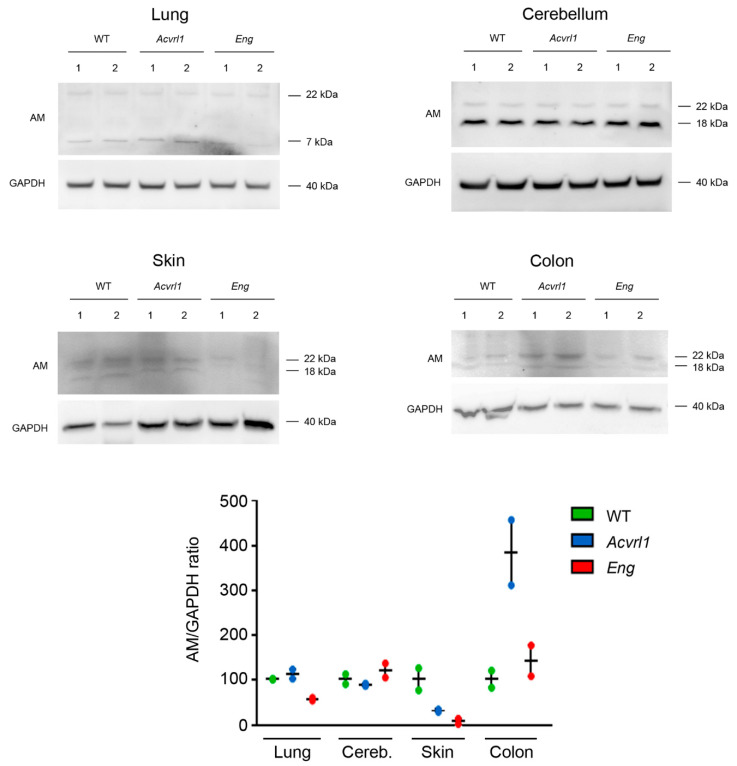
Western blots for AM with protein extracts from the lungs, cerebellum, skin, and colon of the three genotypes. GAPDH was used as a housekeeping protein. The AM/GAPDH ratio was calculated and the data were standardized to the WT expression. See also Supplementary Materials, Figures S1–S4 and Tables S1–S4.

In order to confirm the immunohistochemical results, Western blotting was performed in selected tissues (Figure 10). Previous publications showing Western blots for AM have shown that antibodies may detect up to three specific AM immunoreactive bands corresponding to the complete pre-pro-hormone (~22 kDa), an intermediate (~18 kDa), and the mature peptide (~7 kDa) [25]. Depending on the specific processing program and release rate of each organ, some bands may not appear in the blots. In our case, the lung showed two bands of approximately 22 kDa and 7 kDa, indicating that the mature peptide was temporarily stored in the pulmonary cells. In contrast, the cerebellum, skin, and colon had the 22 kDa and 18 kDa bands, but lacked the smallest one, suggesting that, in these organs, the mature peptide is released to the bloodstream as soon as it is generated. Although the small number of samples precluded any statistical analysis, we can see a trend towards a reduction in the amount of AM in the skin of both mutants, and a clear increase in the expression on the colon of Acvrl1 animals (Figure 10 and Supplementary Materials, Figures S1–S4 and Tables S1–S4).

## 4. Discussion

In this study, we found that AM expression is not affected by mutations in the *Eng* and *Acvrl1* genes in a consistent pattern. On the contrary, in some organs (lungs, stomach, pancreas, heart, kidneys, ovaries, brain cortex, hippocampus, foot skin, and microvessels) there were no significant changes, whereas, in others, we found either reduced expression (fat, skin, and adrenals) or enhanced production of AM (cerebellum and colon). In addition, we found no differences in the circulating levels of AM among genotypes. These results contradict our initial hypothesis that the increased AM expression found in HHT patients [10] may be due directly to the mutations of these patients in the *ENG* or *ACVRL1* genes, but seem to suggest a very complex interplay between the AM system and the TGF-β family of peptides and receptors, which includes BMP-9 [26].

This study has some limitations. First, the number of animals was low and we may have missed additional differences that may have become evident with a higher sample number. In addition, although we selected organs with high AM expression [16], there are many other organs we did not collect that may have had some more exciting information on the regulation of AM by the *Eng* or *Acvrl1* genes.

The only previous reference linking BMP-9 levels to AM expression was published by Tu et al. [27]. In that paper, it was shown that the exposure of human endothelial cells to recombinant BMP-9 resulted in a significant decrease in AM, whereas the endothelial cells of mice lacking BMP-9 had higher levels of AM when exposed to hypoxia. Based on this study, we should had expected a general increase in AM expression in the blood and all organs of mice with faulty BMP-9 receptors, but our results point to a more complex scenario. Something unexpected also happened when the AM levels were studied in TGF-β null mice. Surprisingly, the AM levels were lower in the knockout mice compared to the wild-type ones during intrauterine life, but became elevated after birth [28]. These few reports suggest that the interrelationships among AM, BMP-9, and the other members of the TGF-β family of peptides deserve deeper study, especially if we want to better understand blood vessel physiology in diseases such as HHT.

We found that AM expression did not change in the stomach or pancreas of mutant mice, but was significantly elevated in the colon of *Eng* heterozygotes at the mRNA level, and in the *Acvrl1* mutants at the protein level. AM has been shown to play a protective role in the colon, modulating the microbiota [29], reducing inflammatory bowel disease symptoms [30], and delaying colitis-associated colon cancer progression [21], among other positive functions [31]. Therefore, our results suggest that BMP-9 and its receptors may be also involved in all these protective activities. This field merits further research.

We also found that *Acvrl1* mutant mice expressed significantly lower levels of AM in their fat than either the *WT* or the *Eng* mutant mice. AM is present in the adipose tissue of mice and humans and its expression increases with obesity [32]. AM seems to play a positive role in this location by stimulating lipolysis [33] and reducing inflammation in white adipose tissue [34]. BMP-9 is known to regulate the energy balance in vivo; however, the specific molecular mechanism is still unknown [35]. Our results point out that the Alk1 component of the receptor may be the responsible party for all of these interesting physiological implications.

We paid special attention to the skin, since it is one of the main targets of HHT. The skin is a very heterogeneous organ, and different regions are characterized by different permeability, microbiota, hair and gland contents, etc. [36]. Therefore, we chose three different skin regions in the mice, including the skin of the dorsal area, the ventral area, and the skin covering the feet, which lacks hair. As expected, AM expression was also heterogeneous, with no changes in the foot skin, but with a clear decrease of AM mRNA expression in the dorsal and ventral skin of the *Acvrl1* mutants, and a reduction in protein for both mutants. In the skin, AM is produced by the keratinocytes and all the skin glands in large amounts, and it has been described as a major component of sweat [37]. It contributes to skin regeneration, wound healing, and has antimicrobial activity, thus efficiently contributing to the barrier function of the skin and other bodily surfaces [37]. BMP-9 is also a positive regulator of skin wound healing [38]. According to our results, both the Alk1 and Eng components of the receptor may be involved in this activity.

In the central nervous system, we found no changes in AM expression in the cortex or the hippocampus, but there was a significant increase in AM mRNA in the cerebellum of *Eng* mutants, although this change was not observed at the protein level. AM and its receptors are widely expressed in many areas of the brain [39], indicating that AM plays important roles as a neuromodulator. Using AM conditional knockout mouse models, several behavior-regulating roles have been assigned to AM, including the regulation of equilibrium, anxiety, stress, and pain processing, among others [40,41]. The enhanced expression of AM mRNA in the cerebellum of the *Eng* mutants suggests that BMP-9, acting through the endoglin component of its receptor, may have some impact on the behavioral functions mediated by the cerebellum, including equilibrium maintenance and other cognitive functions. So far, no connections have been reported between the BMP-9 system and behavior modifications.

Another interesting observation was the significant decrease in the AM levels in the adrenals of *Eng* mutants. AM received its name because it was first isolated from a pheochromocytoma, a tumor of the adrenal gland [11]. Although pheochromocytomas arise from the adrenal medulla, AM expression occurs both in the adrenal cortex and the medulla [42]. Together with other adrenal hormones, AM is a major regulator of blood pressure and vascular integrity [16], regulates nitric oxide release [43], and is involved in many cardiovascular and cerebrovascular conditions [17,44]. It has been demonstrated that BMP-9 acts directly on smooth muscle cells, inducing vasoconstriction [45]. Also, the *Eng* and *Acvrl1* mutants have modified levels of nitric oxide [8,9], indicating a significant role of BMP-9 in regulating blood pressure. Our results suggest that the BMP-9 receptor, and especially the endoglin component, may also regulate the synthesis of AM, a potent vasodilator, in the adrenals, thus contributing to a complex regulation of these functions.

The fact that haploinsufficiency for one of the receptor components does not have the same effect as the other was unexpected, but is in line with clinical observations showing that patients with mutations in the *ENG* gene have different symptoms to those whose *ACVRL1* gene is mutated [5]. The reasons for this discrepancy are not currently understood, but our data point to a possible involvement of diverse AM expression due to loss of function for either protein. Obviously, studies with human samples will be needed to confirm this hypothesis.

Our initial goal was to investigate whether mutations in the BMP-9 receptor components may explain the higher levels of AM observed in HHT patients, and specifically in the telangiectases [10]. We did not find any major change in the expression of AM in the blood vessels of the mutant animals, suggesting that the elevated AM levels found in the patients’ telangiectases may be due to inflammation and/or blood vessel damage concomitant to the disease, rather than to a direct relationship with the mutations.

## 5. Conclusions

We have shown that the relationships between AM and the BMP-9 receptor components are rather complex and context-dependent. Our results open intriguing questions about potential phenotypic manifestations of *Eng* and *Acvrl1* mutants that have not yet been studied and that may offer, in the future, a new focus for research on HHT patients.

## Figures and Tables

**Table 1:** Primers used for quantitative real-time PCR.

Gene of Interest	Sense Primer	Antisense Primer	Annealing Temp.
*Adm*	TTGGGTTCACTCGCTTTCCT	TTAGCGCCCACTTATTCCAC	60 °C
*Gapdh*	CATGTTCCAGTATGACTCCACTC	GGCCTCACCCCATTTGATGT	60 °C

## Data Availability

The data presented in this study are available on request from the corresponding author.

## Supplementary Materials

The following supporting information can be downloaded at: https://www.mdpi.com/article/10.3390/biology11030358/s1, Figure S1: (a) Adrenomedullin antibody and (b) GAPDH antibody in mouse lung; Figure S2: (a) Adrenome-dullin antibody and (b) GAPDH antibody in mouse cerebellum; Figure S3: (a) Adrenomedullin antibody and (b) GAPDH antibody in mouse skin; Figure S4: (a) Adrenomedullin antibody and (b) GAPDH antibody in mouse colon; Table S1: Band quantification in mouse lung; Table S2: Band quantification in mouse cerebellum; Table S3: Band quantification in mouse skin; Table S4. Band quantification in mouse colon.
