# Supplementary material for: Endoglin and Activin Receptor-like Kinase 1 (Alk1) Modify Adrenomedullin Expression in an Organ-Specific Manner in Mice
Source: Biology (Basel). 2022 Feb 24;11(3):358. doi: 10.3390/biology11030358 (PMC8945164; doi:10.3390/biology11030358)
Supplement: Supplementary file 1 [file biology-11-00358-s001.zip › biology-1546244-supplementary.pdf]

Endoglin and activin receptor-like kinase 1 (Alk1) modify adrenomedullin expression in an organ-specific manner in mice

Josune García-Sanmartín[1], Judit Narro[1], Alicia Rodríguez-Barbero[2], Alfredo Martínez[1,*]

[1] Angiogenesis Unit, Oncology Area, Center for Biomedical Research of La Rioja (CIBIR), Piqueras 98, 26006 Logroño, Spain.

[2] Vascular Endothelium Pathophysiology (ENDOVAS) Unit, Department of Physiology and Pharmacology, University of Salamanca, and Biomedical Research Institute of Salamanca (IBSAL). Edificio Departamental, Campus Miguel de Unamuno, 37007, Salamanca, Spain.

* Correspondence: amartinezr@riojasalud.es

Supplementary material

1. Original Western blot figures

Figure S1: (a) Adrenomedullin antibody and (b) GAPDH antibody in mouse lungs.

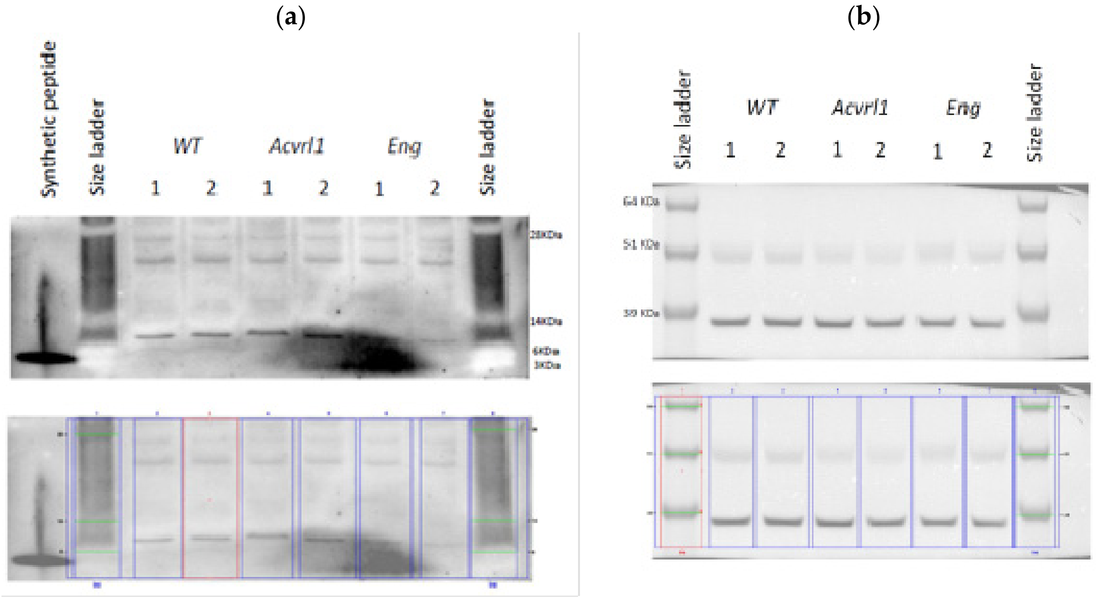

Table S1: Band quantification in mouse lungs.

	WT 1	*WT* 2	*Acvrl1* 1	*Acvrl1* 2	*Eng* 1	*Eng* 2
AM	2544.76	2181.66	2596.23	2804.28	990.04	1295.54
GAPDH	275428638	231912798	275797670	248280662	198631020	232202046
AM/GAPDH	9.2393E-06	9.4072E-06	9.4135E-06	1.1295E-05	4.9843E-06	5.5794E-06

ratio						
AM/GAPDH ratio (%)	99.1	100.9	101.0	121.1	53.5	59.8

Figure S2: (a) Adrenomedullin antibody and (b) GAPDH antibody in mouse cerebellum.

(**a**) (**b**)

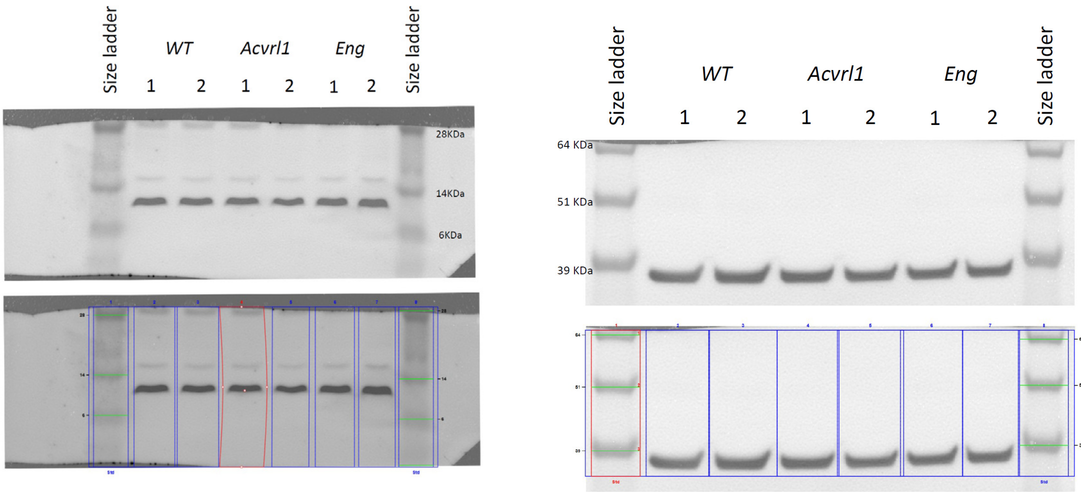

Table S2: Band quantification in mouse cerebellum.

	***WT* 1**	***WT* 2**	***Acvrl1* 1**	***Acvrl1* 2**	***Eng* 1**	***Eng* 2**
AM	20961600	22343296	16939201	20045091	20574040	25328705
GAPDH	463903784	399667554	393104299	437719666	393871494	373397486
AM/GAPDH ratio	4.52E-02	5.59E-02	4.31E-02	4.58E-02	5.22E-02	6.78E-02
AM/GAPDH ratio (%)	89.4	110.6	85.3	90.6	103.4	134.2

Figure S3: (a) Adrenomedullin antibody and (b) GAPDH antibody in mouse skin.

(**a**) (**b**)

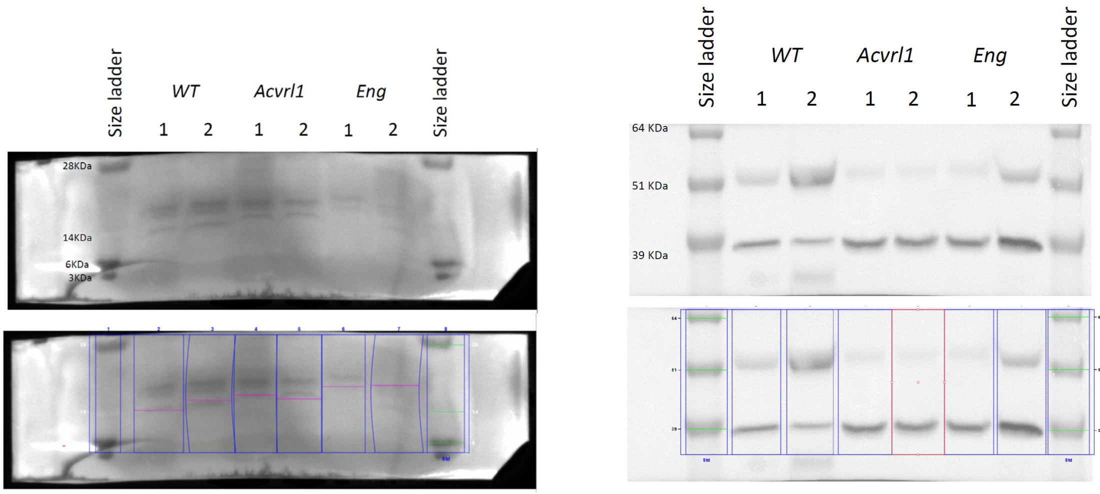

Table S3: Band quantification in mouse skin.

	***WT* 1**	***WT* 2**	***Acvrl1* 1**	***Acvrl1* 2**	***Eng* 1**	***Eng* 2**
AM	8215.91	7548.03	4197.45	5164.85	606.94	2032.78
GAPDH	267968101	151478424	362609134	374480248	570241848	352151835
AM/GAPDH ratio	3.066E-05	4.9829E-05	1.1576E-05	1.3792E-05	1.0644E-06	5.7725E-06
AM/GAPDH ratio (%)	76.2	123.8	28.8	34.3	2.6	14.3

Figure S4: (a) Adrenomedullin antibody and (b) GAPDH antibody in mouse colon.

(**a**) (**b**)

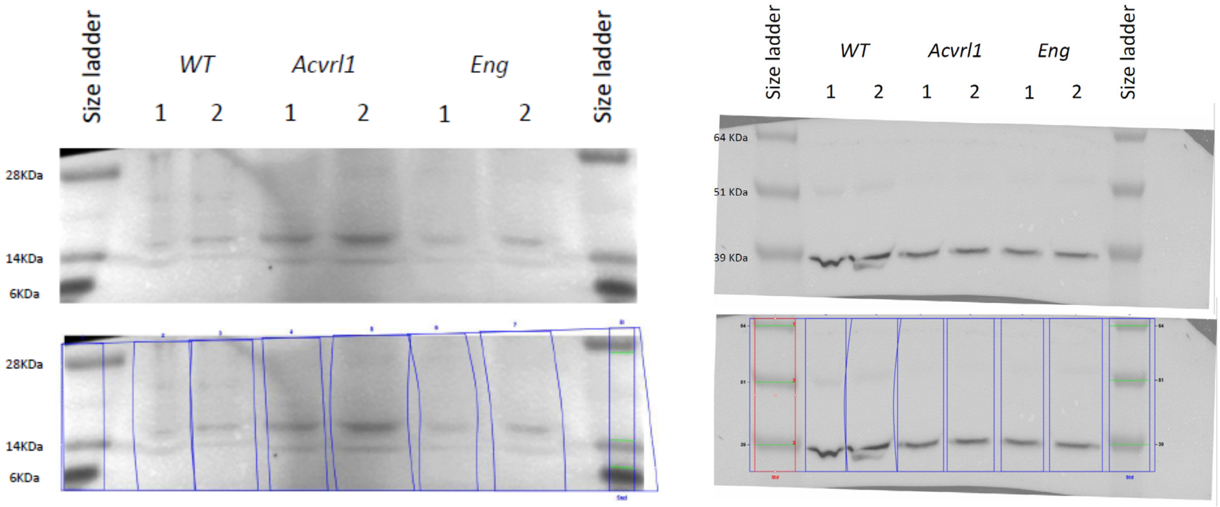

Table S4. Band quantification in mouse colon.

	WT 1	*WT* 2	*Acvrl1* 1	*Acvrl1* 2	*Eng* 1	*Eng* 2
AM	1317.88	1546.3	3011.27	4159.36	918.55	1643.13
GAPDH	251334846	203091180	154341714	145374968	134325920	147515670
AM/GAPDH ratio	5.24E-06	7.61E-06	1.95E-05	2.86E-05	6.84E-06	1.11E-05
AM/GAPDH ratio (%)	81.6	118.4	303.5	445.0	106.4	173.3
